# Alternative Mechanisms to Initiate Translation in Eukaryotic mRNAs

**DOI:** 10.1155/2012/391546

**Published:** 2012-02-16

**Authors:** Encarnación Martínez-Salas, David Piñeiro, Noemí Fernández

**Affiliations:** Centro de Biología Molecular Severo Ochoa, Consejo Superior de Investigaciones Científicas, Universidad Autónoma de Madrid, Nicolás Cabrera, 1, Cantoblanco, 28049 Madrid, Spain

## Abstract

The composition of the cellular proteome is under the control of multiple processes, one of the most important being translation initiation. The majority of eukaryotic cellular mRNAs initiates translation by the cap-dependent or scanning mode of translation initiation, a mechanism that depends on the recognition of the m^7^G(5′)ppp(5′)N, known as the cap. However, mRNAs encoding proteins required for cell survival under stress bypass conditions inhibitory to cap-dependent translation; these mRNAs often harbor internal ribosome entry site (IRES) elements in their 5′UTRs that mediate internal initiation of translation. This mechanism is also exploited by mRNAs expressed from the genome of viruses infecting eukaryotic cells. In this paper we discuss recent advances in understanding alternative ways to initiate translation across eukaryotic organisms.

## 1. Alternative Translation Initiation Mechanisms: An Important Layer of Gene Expression Control

The coding capacity of eukaryotic genomes is much larger than anticipated. Many layers of gene expression control operate at the posttranscriptional level, as illustrated by the RNA splicing process, the noncoding RNAs regulatory elements, and the large repertoire of factors that contribute to control mRNA transport, localization, stability, and translation. Translation control is one of the posttranscriptional cellular processes that exert a profound impact on the composition of the cellular proteome. This is particularly relevant to maintain homeostasis in response to stress induced by a large variety of environmental factors, as well as during development or disease [1]. In addition, these layers of gene expression control contribute to increase the coding capacity of the genome by generating different polypeptides from the same transcriptional unit.

**Figure 1:**
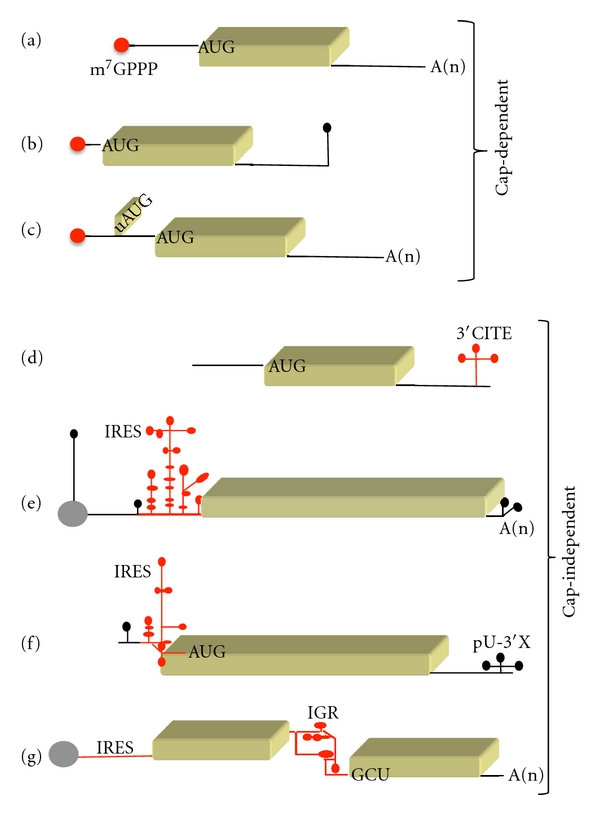
Schematic representation of eukaryotic mRNAs. (a) Features of a conventional mRNA. The red circle at the 5′end depicts the cap (m^7^Gppp); A(n) depicts the poly(A) tail at the 3′end. (b) (c) Schematic of atypical RNA structures, stem loops (black hairpin), or uAUGs, respectively, located in mRNAs translated via cap-dependent initiation. (d) RNA structural elements located in the 3′untranslated region of viral RNAs mediating cap-independent translation (3′CITE). Different types of IRES elements found in the viral RNA of picornaviruses (e), hepatitis C (f), and dicistroviruses (g) are schematically depicted in red.

The majority of cellular mRNAs initiate translation by a mechanism that depends on the recognition of the m^7^G(5′)ppp(5′)N structure (termed cap) located at the 5′end of most mRNAs (Figure 1(a)). This manner of initiating translation involves a large number of auxiliary proteins termed eukaryotic initiation factors (eIFs) [1]. The 5′cap structure is recognized by eIF4E that, in turn, is bound to the scaffold protein eIF4G and the RNA helicase eIF4A (within a trimeric complex termed eIF4F). Additionally, eIF4G further interacts with eIF3 and the poly(A)-binding protein (PABP) that is bound to the poly(A) tail of the mRNA. Separately, the 40S ribosomal subunit associates with the ternary complex (TC) consisting of the initiator methionyl-tRNA_i_ and eIF2-GTP, leading to the formation of the 43S complex that is stabilized by eIF1A and eIF3. Following assembly of the 43S complex into the eIF4F-bound mRNA, scanning of the 5′UTR region commences until the first AUG codon is encountered, leading to the formation of the 48S initiation complex. At this step, eIF1 is displaced and eIF5 mediates the hydrolysis of eIF2-bound GTP; joining of the 60S subunit is then mediated by eIF5B yielding the 80S ribosome that gives rise to the start of polypeptide synthesis. For a review on the translation initiation process, see [1] and references therein.

Various alternative mechanisms to initiate translation are, however, operative during cellular stress [1, 2]. Furthermore, atypical mRNAs that lack the cap structure at the 5′end or the poly(A) tail at the 3′end employ specific mechanisms to initiate translation. Histones are among the most abundant proteins in eukaryotic cells, despite having mRNAs with an organization that is incompatible with the conventional scanning initiation model. Peculiar features of metazoan histone mRNAs are that they harbor a short 5′UTR and lack a poly(A) tail. Instead, these mRNAs harbor a conserved stem loop near the 3′end (Figure 1(b)) that is recognized by the stem-loop-binding protein (SLBP). In addition, the open reading frame of mouse histone mRNA contains two structural elements critical for translation initiation. One of them binds to eIF4E without the need of the cap, such that the 43S complex is recruited to this site and loaded near the AUG start codon [3]. This process is assisted by a second structural element located downstream of the initiator triplet that sequesters the cap, facilitating the direct loading of the ribosome on the cognate codon.

A different example of unconventional RNA organization is presented by a plant viral RNA, the pea enation mosaic virus, that initiates translation using a cap-independent mechanism. This mRNA does not possess a cap at the 5′end even though it requires eIF4E for translation initiation. In this particular case, the RNA structure adopts a pseudoknot structure that projects a guanosine residue into the cap-binding pocket of eIF4E [4].

In addition to the 5′-cap and 3′-poly(A) tail, eukaryotic mRNAs can contain upstream open reading frames (uORFs) (Figure 1(c)), 3′cap-independent translation elements (3′CITEs) (Figure 1(d)), or internal ribosome entry site (IRES) elements (Figures 1(e), 1(f), and 1(g)). These types of structural elements can act as sensors of environmental factors, mediate efficient translation of some viral mRNAs, or control translation of mRNAs that encode proteins performing critical roles during cell death, DNA-damage response, or in the differentiation process of higher eukaryotes and algae [5–10]. In general, these structural elements act as strong barriers to scanning ribosomes in the 5′UTR of mRNAs. Hence, their presence is incompatible with the scanning model of translation initiation, and the corresponding mRNAs have evolved different manners to initiate translation using nonconventional mechanisms.

One extensively analyzed uORF-containing mRNA is that encoding the yeast transcription factor GCN4. Translation of this particular mRNA is strongly enhanced during nutrient deprivation, an event that induces eIF2*α*-phosphorylation leading to low levels of TC and, thus, inhibiting cap-dependent translation. However, GCN4 mRNA overcomes the translation inhibitory effects of four uORFs under low TC levels by allowing a fraction of posttermination 40S subunits to reinitiate at the authentic GCN4 start codon [1]. In mRNAs subjected to polyamine-responsive translation regulation, uORFs act as sensors of polyamine levels [10]. Finally, in many other mRNAs, uORFs are obstacles that block or delay scanning ribosomes causing a strong reduction of protein synthesis, as exemplified in the 5′ terminal region of p53 mRNA or the embryonic form of the chicken proinsulin mRNA [5, 6].

Consistent with the fact that mRNA translation operates on functionally circularized molecules, regulatory elements also are located at the 3′UTR; this is the case of 3′CITE elements which are particularly abundant in plant RNA viruses [7, 11, 12] and promote cap-independent translation by means of complex RNA structures that generate functional bridges between the 5′ and 3′UTRs of the mRNA.

In summary, translation initiation mechanisms affecting the efficiency of protein synthesis of a given mRNA are diverse and, importantly, more frequent than anticipated, sometimes giving rise to the expression of different polypeptides from a single transcriptional unit. Therefore, presence of any of these regulatory elements can seriously complicate efforts to accurately define the sites of translation initiation at the genomewide scale.

## 2. IRES Elements in Eukaryotic Organisms and Their Viruses

IRES elements are specialized RNA regulatory sequences governing cap-independent translation initiation in eukaryotic mRNAs that are translated during cellular stress, that is, when cap-dependent translation is compromised [2, 13, 14]. IRES elements, initially reported in the genomic RNA of two picornaviruses (namely, poliovirus (PV) and encephalomyocarditis virus (EMCV)), drive internal initiation of translation in the mRNA of all members of the *Picornaviridae* family [15–20] (Table 1). Soon after their discovery, IRES elements were also found in other RNA viruses infecting mammals, such as hepatitis C (HCV), pestiviruses [21, 22], or retroviruses [23–27], as well as in RNA viruses infecting invertebrates [28–33], plants [34–39], and protozoa [40, 41]. Recently, IRES-dependent translation in mRNAs transcribed from DNA viruses belonging to the *Herpesviridae* family has been reported [42–45] (Table 1).

As expected from the fact that viruses require components of the host machinery to translate their genome, IRES elements have been found in mRNAs encoded in the genome of the host (Table 2). Thus, IRES-dependent translation initiation has been described in mRNAs expressed in animal cells, both vertebrate and invertebrate [46–69], plants, and yeasts [70–73]. Not surprisingly, various examples of IRES elements reported in animal cells, plants, or yeasts drive internal initiation of translation in mRNAs that encode proteins performing similar functions or belonging to the same regulatory pathway, for example, nutrient deprivation, apoptosis, or heat-shock (see Table 2). Published IRES elements are available at (http://iresite.org/) (http://140.135.61.9/ires/) [74, 75].

The ability of being translated under conditions inhibitory to cap-dependent initiation, though with different efficiency, is a general feature of all IRES-containing mRNAs. With notable exceptions [23, 37], IRES elements are located in the 5′UTR of mRNAs upstream of the initiator codon. Other features such as long length of the 5′UTR (200 to 500 nucleotides), heavy RNA structure, high GC content, initiation at non-AUG codons, ignored AUGs upstream of the functional start codon are often but not universally found [46, 53, 76, 77]. For instance, some IRES elements found in plant RNA viruses, *D. melanogaster* and *S. cerevisiae,* have a high AU content [37, 62, 63, 73]. In this regard, the 5′UTR sequences of mRNAs are highly divergent [78]. This is consistent with the great sequence diversity of the currently known IRES elements (http://rfam.sanger.ac.uk/) found in mRNAs of different organisms, including viruses, protozoa, yeast, plants, and animals. Furthermore, this lack of sequence conservation creates serious problems to predict the presence of IRES elements in eukaryotic mRNAs using computational methods. Therefore, defining critical hints (short primary sequences, tertiary structure elements, unique RNA-binding protein motifs, etc.) of model IRES elements is crucial to accurately predict putative IRES elements at the genomic level.

## 3. Types of IRES Elements

Two picornavirus RNAs, PV and EMCV, contain the first reported IRES elements [15, 16]. This property was later extended to all picornavirus RNAs as well as to several positive-strand RNA viruses, such as HCV, pestiviruses, and dicistroviruses [79]. Nevertheless, it is remarkable that despite performing the same function, viral IRES elements differ in nucleotide sequence, RNA secondary structure, and trans-acting factors requirement. A distinctive feature of the picornavirus IRES is their long length that varies between 350 to 450 nucleotides, depending on the virus genera. Furthermore, picornavirus IRES elements are classified in four types (termed I, II, III, and HCV-like) according to their RNA structure organization. The genome of picornaviruses consists of a single-stranded RNA of positive polarity that harbors a short poly(A) tail at the 3′end (Figure 1(e)). However, picornavirus RNAs differ from cellular mRNAs in having a long, heavily structured 5′UTR and a viral-encoded protein (VPg) covalently linked to the 5′end instead of cap, hence, incompatible with the cap-dependent mechanism of translation initiation. Not surprisingly, translation of the viral genome is governed by the IRES element using a cap-independent mechanism that is resistant to the action of viral proteases. Picornavirus-encoded proteases execute the processing of the viral polyprotein but also recognize as substrates several host factors. Among the host factors proteolyzed during infection are eIF4G and PABP [80, 81], which are key components of the cap-dependent translation initiation machinery. Thereby, cleavage of host factors induces the shut-off of cap-dependent translation in infected cells.

Picornavirus elements IRES belonging to types I and II require the C-terminal end of eIF4G, eIF4A, and eIF3 to assemble 48S initiation complexes [82–84]. Type III IRES require intact eIF4G, and, in contrast, the HCV-like IRES does not need eIF4G to assemble 48S complexes [18]. In addition to eIFs, auxiliary factors termed IRES transacting factors (ITAFs) contribute to modulate (either stimulate or repress) picornavirus IRES activity. In support of the relevance of factors different than eIFs for internal initiation, transcripts encompassing the region interacting with eIFs do not possess IRES activity [85], indicating that interaction with eIFs is necessary but not sufficient for IRES function.

The HCV viral RNA does not possess poly(A) tail; instead, a poly(U) tract and a complex RNA structure are located near the 3′end (Figure 1(f)). The HCV IRES element is located close to the 5′end of the viral genome and differs profoundly in RNA structure organization from picornavirus IRES belonging to types I, II, and III. Specifically, the HCV IRES (340 nucleotides) is arranged in structural domains II, III, and IV, including a pseudoknot upstream of the AUG start codon [86] which is conserved with pestivirus IRES elements [22]. Domain III participates in the interaction with eIF3 and the 40S subunit, while domain II helps to accommodate the mRNA in the tRNA-exit site of the ribosome and mediates eIF2 release during 80S assembly [87, 88]. Interestingly, HCV-like IRES elements with similar eIF requirement have been found in some picornavirus genera [18, 89, 90], which presumably arose by recombination events.

A unique type of IRES element is located in the intergenic region (IGR) of the genome of dicistroviruses (Figure 1(g)). This RNA region spans about 200 nucleotides and adopts a tertiary structure including three pseudoknots that functionally substitute the initiator tRNA during internal initiation [29, 91]. The IGR mimics a tRNA anticodon loop base-paired to mRNA and a translation elongation factor, facilitating initiation without the help of eIFs. A unique feature of these IRES elements is to initiate protein synthesis at non-AUG codons (CUU, GCU, CCU, CUC, depending on the dicistrovirus genus) [92, 93], with preference for alanine-coding triplets.

IRES elements were reported in various cellular mRNAs that remained attached to polysomes under conditions inhibitory to cap-dependent translation [51, 94]. These mRNAs contain a cap at the 5′end although they are translated at very low levels and have the capacity to switch to an IRES-dependent mechanism when cap-dependent initiation is impaired. This process is assisted by ITAFs, a group of RNA-binding proteins that are thought to help in the proper folding of the IRES region facilitating the mRNA recruitment to the translation machinery.

Thus, attending to the essential requirements for internal initiation, IRES elements can be grouped in two main categories: (a) those that do not need proteins to assemble the initiation complex (e.g., the IGR of dicistroviruses that adopts a docking structure capable of fitting in the ribosomal subunit [95]) and (b) those that do need factors to recruit the ribosome (typically, picornaviruses, HCV, and cellular IRES elements [60, 96, 97]). Within the second category, distinct groups can be made depending on the RNA structural motifs and proteins required for activity.

## 4. RNA Structural Motifs Found in IRES Elements

RNA structure plays a fundamental role in viral IRES-dependent translation initiation [13]. In support of this, mutations leading to the disruption of specific RNA structure motifs impaired IRES activity while the corresponding compensatory mutations restored IRES function [21, 98]. Furthermore, RNA structure of viral IRES elements is organized in modules which are phylogenetically conserved [99–102], providing evidence in favour of a distribution of functions among the different RNA domains [103, 104].

Examples of structural motifs found in IRES elements are the pseudoknots (Pks). These are tertiary motifs that play important role in the IRES of HCV, bovine viral diarrhea virus (BVDV), and classical swine fever virus (CSFV) [22]. Three different Pks conform the IGR of cricket paralysis virus (CrPV) and plautia stali intestine virus (PSIV), as well as other dicistroviruses [91, 93]. Giardiavirus (GLV) and tobacco etch virus (TEV) IRES elements also contain Pk structures [39, 40]. While these IRES elements are located in genetically distant RNA viruses, the Pk structure is conserved, indicating that the RNA organization is biologically relevant for internal initiation. Some cellular IRES elements were reported to contain Pk structures, as illustrated by c-myc and L-myc [77, 105]. In further support of the role of RNA structure for IRES-dependent translation, the zipper model proposed for the cationic amino acid transporter CAT-1 mRNA suggested that RNA structure modification via translation of an upstream uORF induces the formation of the active IRES [46].

Evidence for tight links between RNA structure and biological function are provided by the conservation of structural motifs within IRES elements of highly variable genomes. Specifically, the purine-rich GNRA and RAAA motifs (N stands for any nucleotide, and R, purine), as well as the G : C-rich stems that hold these motifs are conserved between aphthovirus and cardiovirus [106, 107]. In the case of foot-and-mouth disease virus (FMDV), the prototype of the aphthovirus genus, the central IRES domain is a self-folding region that has been proposed to instruct the functional conformation of the whole IRES element [85, 108, 109]. Along this idea, RNA structural analysis provided evidence for stem loops whose structural conformation depends on distant interactions within this domain, involving residues of the GNRA motif [110]. Thus, it is likely that RNA structural motifs located in the apical region of the central domain could constitute a signature of picornavirus type II IRES elements.

The GNRA motif of picornavirus IRES adopts a tetraloop conformation at the tip of a stem loop [111–113]. This motif is essential for IRES activity in FMDV and EMCV [114, 115], showing a strong preference for GUAA in the case of the FMDV IRES [108]. This observation, together with the lack of genetic variability within the apical stem and the covariation observed in the adjacent stems, pointed towards their joint contribution to IRES activity. Importantly, mutational analysis of the invariant apical stem revealed a better performance of G : C than C : G base-pairs, demonstrating the relevance of the three-dimensional RNA conformation for IRES activity [106].

Although related RNA viruses share the overall organization of their genomic RNA, viral IRES is organized in high-order structures that differ between distant families. Cryo-electron microscopy studies of the HCV IRES and the IGR provided information on the capacity of these RNAs to be accommodated in the interface of the ribosomal subunits [88, 95]. Even though the IGR of CrPV and the HCV IRES exhibit different structural organization, they interact with the ribosomal protein RpS25 [116] and induce similar conformational changes in the 40S ribosomal subunit. This finding opens the possibility that IRES elements could possess a universal structural motif mediating its direct interaction with the 40S subunit. This putative universal RNA motif still remains elusive, but it could be a promising tool to search for unidentified IRES elements at the genomic level.

Concerning the identification of structural motifs conserved between genetically distant RNAs, the IRES region of FMDV, EMCV, CrPV, HCV, CSFV, and BVDV contains a structural element recognized as substrate of the RNase P ribozyme [117–119]. RNase P is a nuclear structure-dependent endonuclease involved in the processing of the tRNA precursor, that also recognizes as substrate viral RNAs containing tRNA-like structures at the 3′end. The RNase P cleavage site in the FMDV IRES maps within an internal region that is involved in tertiary interactions; in addition, defective IRES mutants bearing modified RNA structures exhibited a differential response to ribozyme cleavage both *in vitro* and in transfected cells [119, 120]. The significance of the RNase P recognition motif in IRES elements is unknown since there is no proof for its direct involvement in the translation process. However, it does not constitute an RNA processing motif in transfected cells [121], consistent with the fact that the picornavirus infection cycle, as well as that of HCV and pestivirus, occurs in the cytoplasm of infected cells; therefore, the viral RNA has no access to RNase P.

The possibility that this structural motif constitutes a remnant of an ancient tRNA-like structure, similar to that found in the IGR IRES, is open to further investigations. Indeed, the evolutionary origin of IRES elements is unknown, but it has been proposed that this mode of initiating protein synthesis could be operating earlier than the cap-dependent [122]. In keeping with this hypothesis, the IRES property of self-interacting with the ribosome is a very attractive idea, also consistent with the finding that IRES activity is sensitive to changes in ribosome composition [123–126].

Another possibility to explain the presence of tRNA-like motifs within viral IRES elements is that they were inherited from RNA replication signals accommodated to assist in the translation process. In plant RNA viruses, tRNA-like structures located at the 3′end of the viral genome control cap-independent translation initiation and viral RNA replication [11, 127]. RNA-RNA interactions between the 3′and the 5′UTR of the viral genome assist in these processes. In this regard, long-range RNA-RNA interactions between the 5′ and the 3′end of some viral genomes have been observed [128, 129]. Consistent with a functional link between the ends of the viral RNA, IRES activity is stimulated by the 3′UTR [130, 131]. In picornavirus RNAs, the 3′UTR is composed of two stem loops and a short poly(A) tail that are required for replication and infectivity [132]. Furthermore, the insulin-like growth factor II mRNA-binding protein 1 (IGF2BP1) was identified among the proteins identified in complexes assembled with RNAs that contained the HCV IRES and the 3′UTR. This protein coimmunoprecipitates with eIF3 and the 40S subunit [133], suggesting that it enhances HCV IRES activity by recruiting the ribosomal subunits to a pseudo circularized RNA. Thus, bridging of 5′ and 3′ends involves direct RNA-RNA contacts and RNA-protein interactions. These results provide a mechanistic basis for translation stimulation and replication of the viral RNA resembling the synergistic stimulation of cap-dependent translation.

## 5. RNA-Protein Interactions Controlling IRES Activity

The lack of conserved features among distantly related IRES elements has led to the view that different IRES elements could recruit the ribosomal subunits assisted by unique sets of ITAFs. Along this idea, riboproteomic approaches have facilitated the identification of various proteins interacting with different IRES elements [134]. Most ITAFs are RNA-binding proteins previously identified as transcription regulators, splicing factors, RNA transport, RNA stability, or translation control proteins [133, 135, 136]. Typical examples of multifunctional proteins that act as ITAFs are the polypyrimidine tract-binding protein (PTB), the poly-r(C) binding protein (PCBP2), the SR splicing factor (SRp20), the far upstream element binding protein 2 (FBP2), the lupus La antigen (La), or Gemin5, among others [136–140].

The IRES elements of HCV and HIV-1 differ from those of picornaviruses not only in RNA structure but also in some factor requirement. Assembly of the HCV IRES-48S initiation complex requires eIF3, but not eIF4G [96]. Consistent with this, eIF3 has been identified by mass spectrometry of IRES-bound protein complexes [135, 141]. Other proteins bound to HCV and picornavirus IRES are PTB, PCBP 2, nucleolin, Gemin5, upstream of n-ras (unr), heterogeneous nuclear RNA-binding protein (hnRNP) A1/A2, La autoantigen (La), NS1-associated protein, as well as several RNA helicases DEAH-box polypeptide 9 (DHX9) [134]. Gemin5 binds directly to FMDV and HCV IRES regions and down regulates translation efficiency. Additionally, Gemin5 binds m^7^GTP [142], explaining its down regulation of cap-dependent translation [140]. In contrast, the HIV-1 IRES is stimulated by hnRNP A1/2, the RNA helicase DEAD/H Box  3 (DDX3), the human Rev-interacting protein (hRIP), and the nuclear RNA-binding protein Src-associated in mitosis (Sam68) [143].

Cellular IRES elements are typically present in mRNAs encoding stress response proteins, such as those needed during nutrient deprivation, temperature shock, hibernation, hypoxia, cell cycle arrest, or apoptosis (Table 2) [2, 14, 57, 144]. However, with the exception of polypyrimidine tracts, conservation of primary sequence is not readily detected between viral and cellular IRES elements [145]. The observation that cellular IRES elements do not share overall structural similarity [146] has led to the view that short motifs may control the interaction with transacting factors needed to recruit the mRNA to 40S subunits. In favor of this hypothesis, PTB stimulates the IRES of the apoptotic protease-activating factor 1 (apaf-1), BCL2-associated athanogene (BAG)-1, and the hypoxia-inducible factor (HIF1a) [50, 147], allowing the synthesis of proteins that mediate cell survival under apoptosis, hypoxia, nutrient deprivation, or cell growth dysregulation. Proteins interacting with the lymphoid enhancer factor (LEF-1) IRES recently identified using biotin-tagged RNAs combined with stable isotope labeling with amino acids in cell culture (SILAC)-based quantitative mass spectrometry [65] include the splicing-related protein proline and glutamine-rich SFPQ/PSF, the non-POU domain-containing octamer-binding nuclear RNA-binding protein (nonO/p54nrb), PCBP 2, HuR, and the oncoprotein DEK (named by the initials of a patient affected of acute myeloid leukemia). Investigating whether the proteins identified in intracellular IRES-ribonucleoprotein complexes perform the same or different functions from those found in IRES complexes assembled *in vitro* requires further work.

Since ITAFs usually act in large complexes with various factors within the cellular compartments, proteins interacting with different targets may lead to distinct effects depending on the target RNA and the other partners of the complex. Thus, changes in the abundance, posttranslational modifications, or subcellular location of ITAFs could be responsible for the distinct IRES response to stress conditions. For example, the kinase PITSLREp58 IRES is specifically activated during mitosis, while mPer 1 translation oscillates during circadian rhythmic period [148, 149]; the Apaf IRES is activated during apoptosis, while the X-linked inhibitor of apoptosis protein (XIAP) is inhibited [150]. Relocalization of hnRNP A1 mediates internal initiation of c-myc, unr, cyclin D1, vascular endothelial growth factor (VEGF), fibroblast growth factor (FGF-2), Apaf-1, and XIAP mRNAs [151]. In contrast, death-associated protein (DAP 5), nuclear factor NF45, G-rich RNA sequence binding factor (GRSF-1), fragile-X mental retardation protein (FMRP), dyskeratosis congenita (DKC1), heterogeneous nuclear ribonucleoprotein D-like protein (JKTBP1), or zinc-finger protein (ZNF9) are IRES-specific [57, 61, 152–154]. Hence, individual mRNAs seem to use different mechanisms to evade the global repression of protein synthesis.

## 6. Perspectives towards the Identification of IRES Elements at Genomewide Scale in Eukaryotes

A functional assay testing the cap-independent capacity and the ability to resist cap-inhibitory conditions is the usual way to identify IRES elements in mRNAs. This is a cumbersome task in terms of genomewide scale identification of IRES elements in eukaryotic genomes. To facilitate this task, short conserved structural motifs identified in model IRES elements could provide a tool to search for putative IRES at a genomic level. In considering critical features of IRES elements, signals that may be suggestive of functional IRES could be the presence of polypyrimidine tracts, pseudoknots near the start codon, or hairpin-loops mimicking those present in the IRES of picornavirus, HCV, or the IGR of dicistrovirus RNAs. Although none of these features individually are sufficient to define a functional IRES element, the presence of one (or more) of these motifs may provide hints to select potential IRES in mRNAs.

The question that remains unresolved is what are the distinctive features of IRES elements that may allow their accurate prediction at the genomewide scale, even though computer programs have being designed to predict IRES elements using bioinformatics tools (http://140.135.61.9/ires/) [75]. From our point of view this question is still far from being answered; however, the detailed molecular, biochemical, and structural characterization of model IRES elements will provide critical hints to reveal the presence of similar elements within eukaryotic genomes.

## Figures and Tables

**Table 1:** Distribution of IRES elements in viral mRNAs.

Host	*Virus family*/genus	Virus/IRES name	Reference
Mammals	*Picornaviridae*/Enterovirus	Poliovirus (PV)	[15]
	*Picornaviridae*/Cardiovirus	Encephalomyelitis virus (EMCV)	[16]
	*Picornaviridae*/Aphthovirus	Foot-and-mouth disease virus (FMDV)	[17]
	*Picornaviridae*/Teschovirus	Porcine teschovirus-1 (PTV-1)	[18]
	*Picornaviridae*/Kobuvirus	Aichivirus (AiV)	[19]
	*Picornaviridae*/Senecavirus	Seneca Valley virus (SVV)	[20]
	*Flaviviridae*/Hepacivirus	Hepatitis C virus (HCV)	[21]
	* Flaviviridae*/Pestivirus	Classical swine fever virus (CSFV)	[22]
	*Retroviridae*/Lentivirus	Human immunodeficiency virus-2 (HIV-2)	[23]
	*Retroviridae*/Lentivirus	Human immunodeficiency virus-1 (HIV-1)	[24]
	*Retroviridae*/Retrovirus	Moloney murine leukemia virus (MoMLV)	[25]
	*Retroviridae*/Lentivirus	Feline immunodeficiency virus (FIV)	[26]
	*Retroviridae*/Retrovirus	Mouse mammary tumor virus (MMTV)	[27]
	*Herpesviridae*/Cytomegalovirus	Human cytomegalovirus latency (pUL138)	[42]
	*Herpesv.*/Lymphocryptovirus	Epstein-Barr virus (EBNA-1)	[44]
	*Herpesv.*/Mardivirus	Herpes virus Marek's disease (MDV RLORF9)	[45]
	*Papovaviridae*/Polyomavirus	SV40 polycistronic 19S (SV40 19S)	[43]

Insects	*Dicistroviridae/*Cripavirus	Rhopalosiphum padi virus (RhPV)	[28]
	*Dicistroviridae/*Cripavirus	Cricket paralysis virus (CrPV)	[29]
	*Dicistroviridae/*Cripavirus	Ectropis obliqua picorna-like virus (EoPV)	[31]
	*Dicistroviridae/*Cripavirus	Plautia stali intestine virus (PSIV)	[32]
	*Dicistroviridae/*Cripavirus	Triatoma virus (TrV)	[33]
	*Dicistroviridae*/Aparavirus	Bee paralysis dicistrovirus (IAPV, KBV)	[30]

Plants	*Comoviridae*/Nepovirus	Black currant reversion virus (BRV)	[34]
	*Tombusviridae*/Carmovirus	Pelargonium flower break virus (PFBV)	[35]
	*Tombusviridae*/Carmovirus	Hibiscus chlorotic ringspot virus (HCRSV)	[38]
	Tobamovirus	Crucifer-infecting tobamovirus (CrTMV)	[36]
	*Luteoviridae*/Polerovirus	Potato leaf roll polerovirus (PLRV)	[37]
	*Potyviridae*/Potyvirus	Tobacco etch virus (TEV)	[39]

Protozoa	*Totiviridae*/Giardiavirus	Giardiavirus (GLV)	[40]
	*Totiviridae*/Leishmaniavirus	Leishmania RNA virus-1 (LRV-1)	[41]

**Table 2:** Distribution of IRES elements in cellular mRNAs.

Organism	Protein function	IRES name	Reference
Mammals	Apoptotic proteins	Apaf-1	[60]
		XIAP	[56]
		HIAP2/c-IAP1	[57]
		DAP5	[58]
		Bcl-2	[61]
	Oncogene	c-myc	[77]
	Amino acid starvation	CAT-1	[46]
	Nutrient signaling	INR	[59]
	Differentiation	LEF-1	[65]
		PDGF2	[66]
	Hypoxia	HIF-1a	[50]
		VEGF	[55]
		FGF2	[53]
	Heat shock	BiP	[51]
		BAG-1	[49]
	Cold shock	CIRP	[48]
	DNA damage response	p53	[47]
		SHMT1	[54]
	Mitosis	PITSLREp58	[50]
		CDK1	[61]

Insects	Apoptotic proteins,	Rpr, hid	[62]
	Heat shock	hsp70	[63]
		grim, skl	
	Homeotic protein	Antennapedia	[64]
	Insulin signaling	dFoxO	[67]
		dInR	[68]
	Alcohol dehydrogenase	Adh-Adhr	[69]

Plants	Heat shock	HSP101	[70]
	Alcohol dehydrogenase	ADH	[71]

Yeast	Nitrogen assimilation	URE-2	[72]
	glucose starvation	GPR1, NCE102	[73]
		YMR181a, MSN1	
		BOI1, FLO8, GIC1	
